# Exploring the association between brain-derived neurotrophic factor (BDNF) levels and longitudinal psychopathological and cognitive changes in Sardinian psychotic patients

**DOI:** 10.1192/j.eurpsy.2022.414

**Published:** 2022-09-01

**Authors:** U. Isayeva, M. Manchia, D. Primavera, L. Deriu, E. Caboni, N. Iaselli, D. Sundas, M. Tusconi, R. Collu, M. Scherma, A. Squassina, D. Congiu, W. Fratta, P. Fadda, B. Carpiniello

**Affiliations:** 1 University of Cagliari, Section Of Psychiatry, Department Of Medical Sciences And Public Health, Cagliari, Italy; 2 University of Cagliari, Division Of Neuroscience And Clinical Pharmacology, Department Of Biomedical Sciences, Cagliari, Italy; 3 Dalhousie University, Department Of Pharmacology, Halifax, Canada; 4 University of Cagliari, Centre Of Excellence “neurobiology Of Dependence”, Cagliari, Italy

**Keywords:** schizophrénia, bdnf, biomarker, Psychosis

## Abstract

**Introduction:**

Schizophrenia spectrum disorders are among the most debilitating mental disorders and evidence on its pathophysiological underpinnings is scant. The brain-derived neurotrophic factor (BDNF) appears to be involved in the pathophysiology of these complex psychiatric disorders.

**Objectives:**

The present study investigates the longitudinal variation of serum BDNF levels in a 24-month observational cohort study of Sardinian psychotic patients (LABSP). This study assessed the variation in BDNF serum levels and its relationship with psychopathological and cognitive changes. Further, we also examined if genetic variations within the BDNF gene could moderate these relationships.

**Methods:**

Every six months 105 LABSP patients were assessed for their BDNF serum levels, as well as for a series of psychopathological, cognitive, and drug-related measures. Four tag single nucleotide polymorphisms (SNPs) within the BDNF gene were selected and analyzed using Polymerase Chain Reaction (PCR). Longitudinal data were analyzed using mixed-effects linear regression models (MLRM).

**Results:**

Analysis showed significantly lower peripheral BDNF levels in psychotic patients with depressive and negative symptoms. BDNF levels were also decreased in patients scoring lower in cognitive measures such as symbol coding and semantic fluency. In addition, Val66Met polymorphism within the BDNF gene significantly moderated the relationship between the severity of negative symptoms and BDNF levels.

**Conclusions:**

Our findings are consistent with previous literature suggesting that peripheral BDNF levels are associated with some cognitive domains and mood disruption in major psychosis. The results also suggest the lack of association between most BDNF genetic variants, except Val66Met polymorphism, with the severity of negative symptoms.

**Disclosure:**

No significant relationships.

